# Relation of Serum Plasmalogens and *APOE* Genotype to Cognition and Dementia in Older Persons in a Cross-Sectional Study

**DOI:** 10.3390/brainsci9040092

**Published:** 2019-04-24

**Authors:** Dayan B. Goodenowe, Vijitha Senanayake

**Affiliations:** Prodrome Sciences Inc., 204-2366 Avenue C N, Saskatoon, SK S7L 5X5, Canada; v.senanayake@prodromesciences.com

**Keywords:** Alzheimer’s disease, dementia, plasmalogen, *APOE*, cognition, cholesterol

## Abstract

Using a community sample of 1205 elderly persons, we investigated the associations and potential interactions between Apolipoprotein E (*APOE)* genotype and serum phosphatidylethanolamine (PlsEtn) on cognition and dementia. For each person, *APOE* genotype, PlsEtn Biosynthesis value (PBV, the combination of three key PlsEtn species), cognition (the combination of five specific cognitive domains), and diagnosis of dementia was determined. *APOE* genotype and PBV were observed to be non-interacting (*p* > 0.05) and independently associated with cognition: *APOE* (relative to ε3ε3:ε2ε3 (Coef = 0.14, *p* = 4.2 × 10^−2^); ε3ε4/ε4ε4 (Coef = −0.22, *p* = 6.2 × 10^−5^); PBV (Coef = 0.12, *p* = 1.7 × 10^−7^) and dementia: *APOE* (relative to ε3ε3:ε2ε3 (Odds Ratio OR = 0.44, *p* = 3.0 × 10^−2^); ε3ε4/ε4ε4 (OR = 2.1, *p* = 2.2 × 10^−4^)); PBV (OR = 0.61, *p* = 3.3 × 10^−6^). Associations are expressed per standard deviation (SD) and adjusted for serum lipids and demographics. Due to the independent and non-interacting nature of the *APOE* and PBV associations, the prevalence of dementia in *APOE* ε3ε4/ε4ε4 persons with high PBV values (>1 SD from mean) was observed to be the same as *APOE* ε3ε3 persons (14.3% versus 14.0%). Similarly, the prevalence of dementia in *APOE* ε3ε3 persons with high PBV values was only 5.7% versus 6.7% for *APOE* ε2ε3 persons. The results of these analyses indicate that the net effect of *APOE* genotype on cognition and the prevalence of dementia is dependent upon the plasmalogen status of the person.

## 1. Introduction

Apolipoprotein E (*APOE*) is the predominant lipoprotein in the brain [1] and is the strongest genetic risk factor for Alzheimer’s disease (AD) [2]. *APOE* has three common genetic variants (ε2, ε3, ε4) from which four allelic combinations make up approximately 95% of the U.S. population [3]. The AD risk gradient observed across the *APOE* genotypes is remarkable in that the percentage of subjects over the age of 60 with AD ranges from ~3% in ε2ε3 to 5% in ε3ε3 to 18% in ε3ε4 to 70% in ε4ε4 subjects. The potential biological mechanisms responsible for the association of *APOE* genotype with AD risk are poorly understood [4,5].

Recent advances in AD research suggest that lipid abnormalities in the brain start in preclinical stages and are linked with neurodegeneration [6,7]. Lipids play critical structural and physiological roles in the brain [8]. Among phospholipids, glycerophospholipids containing a vinyl ether bond called plasmalogens play a disproportionately larger role in maintaining optimal brain function [9,10]. The deficiency of plasmalogen in brain and blood in AD has been reported by numerous researchers including us, and the association of plasmalogen deficiency with AD is well-accepted in the scientific community [6,7,11,12,13,14]. Furthermore, in AD, brain plasmalogen ethanolamine (PlsEtn) levels are reportedly lower than age-matched controls [11,13,14], and low brain levels correlate with low serum levels [13]. Serum PlsEtn levels have been found to correlate with cognition in subjects with AD, including subjects pathologically confirmed by autopsy, and in serum samples collected at time of death in subjects with post-mortem AD pathology [15]. The decline in peroxisomal function is considered, at least, partially responsible for this plasmalogen depletion [15]. Moreover, lower peroxisomal β-oxidation, a key metabolic system involved in PlsEtn biosynthesis, is associated with lower cognition [16,17]. The role of PlsEtn in health and disease has been recently reviewed in detail [12].

Cholesterol [18,19] and brain cholesterol efflux [20] also play a significant role in the pathogenesis of AD. High Density Lipoprotein (HDL)-mediated cholesterol efflux (HDL-MCE) is a key component of cholesterol homeostasis and involves essentially two steps: intracellular cholesterol regulation and export and extracellular HDL binding and transport. Both PlsEtn and *APOE* have been shown to affect this system: PlsEtn deficient cells exhibit disrupted intracellular cholesterol transport and reduced cholesterol esterification which is restored by either genetic or metabolic restoration of PlsEtn [21]; increasing membrane Polyunsaturated fatty acid (PUFA) bearing PlsEtn concentrations increase cholesterol esterification via increased sterol-O-acetyltransferase (SOAT) and decreased total cholesterol [22]; low PlsEtn levels directly affect HDL-MCE, which is restored when PlsEtn levels are increased [23]; the potency of *APOE*-HDL particles to bind and remove cholesterol from neurons is isoform-dependent (ε2 > ε3 > ε4) [20]; synaptic cholesterol levels are isoform-dependent (ε4 > ε3 > ε2) [24,25]; and differential binding affinities of the *APOE* isoforms to the low-density lipoprotein receptor (LDLR) simultaneously affect cortical cholesterol levels and cognitive function [26].

Therefore, both PlsEtn [23] and ApoE [20] seem to play a role in cholesterol homeostasis and efflux, which could be potential mechanisms by which these variables affect AD pathogenesis. Since PlsEtn and ApoE appear to affect membrane cholesterol via independent mechanisms, we hypothesized that if membrane cholesterol dynamics are involved upstream of cognition and AD, then the PlsEtn and ApoE associations with cognition and odds of AD would interact. However, if membrane cholesterol changes and cognition are downstream effects of AD pathology, then no interaction should be observed. To test this hypothesis, we investigated the associations between *APOE* genotype, serum PlsEtn levels, and serum lipids, such as HDL, on cognition and odds of AD in a well-characterized community-based group of 1205 elderly persons enrolled in the Religious Orders Study (ROS) [27] or Rush Memory and Aging Project (MAP) [28].

## 2. Materials and Methods

### 2.1. Participants

Participants were selected from living subjects currently enrolled in the Religious Orders Study (ROS) or Rush Memory and Aging Project (MAP) [27,28] The Religious Orders Study began in 1994 and enrolls older Catholic clergy from across the USA and the Rush Memory and Aging Project began in 1997 and enrolls older lay persons from across northeastern Illinois. Both studies enroll persons without known dementia who agree to annual clinical evaluation. Both studies were approved by the Institutional Review Board of Rush University Medical Center and participants in both studies signed an informed consent. Information on ethics board approvals can be found in reports published by the ROS and MAP study teams [27,29]. The studies were conducted by the same investigative team and have a larger common core that allowed efficient merging of data for analyses [30]. We tested 1205 participants who had *APOE* genotyping data available.

### 2.2. Clinical Evaluation

A uniform structured clinical evaluation was performed to document the level of cognition, and the presence of dementia, AD, mild cognitive impairment (MCI), and other causes of cognitive impairment as previously described [16,31]. Dementia diagnosis was made separately, and a dementia diagnosis includes dementia due to other neurological diseases, in addition to AD. Therefore, not all subjects in the dementia category were included in the AD category. Dementia diagnosis was based on current diagnostic evaluation for dementia according to the Diagnostic and Statistical Manual of Mental Disorders (DSM-III-R) definition of dementia [17]. AD diagnosis was made according to the National Institute of Neurological and Communicative Disorders and Stroke/Alzheimer’s Disease and Related Disorders Association (NINDCS/ADRDA) criteria [16]. The diagnosis of AD required a history of cognitive decline and evidence of impairment in memory and other cognitive abilities. Each study had a battery of 21 tests (Mini-Mental State Examination, Logical Memory Ia, Logical Memory IIa, Immediate story recall, Delayed story recall, Word List Memory, Word List Recall, Word List Recognition, Complex Ideational Material, Boston Naming Test, Category Fluency, National Adult Reading test, Digit Span Forward, Digit Span Backward, Digit Ordering, Symbol Digit Modalities Test, Number Comparison, Stroop word reading, Stroop color naming, Judgment of Line Orientation, and Standard Progressive Matrices); 19 tests (except Mini-Mental State Examination and Complex Ideational Material) were used to create a composite measure of global cognition (GCOG) based on the average z-score using the means and standard deviations from the baseline visit [28]. The tests were also used to summarize cognition into five cognitive domains: episodic memory (Logical Memory Ia, Logical Memory IIa, Immediate story recall, Delayed story recall, Word List Memory, Word List Recall, and Word List Recognition), semantic memory (Boston Naming Test, Category Fluency and National Adult Reading test), working memory (Digit Span Forward, Digit Span Backward and Digit Ordering), perceptual speed (Symbol Digit Modalities Test, Number Comparison, Stroop word reading, and Stroop color naming), and visuospatial ability (Judgment of Line Orientation and Standard Progressive Matrices) [28]. Composite score and scores representing each cognitive domain were used for statistical analyses.

### 2.3. Serum Biomarker Analyses

Fresh serum from a serum separator tube was sent to Quest Diagnostics (Chicago, IL) for HDL-Cholesterol (HDL-C), Low Density Lipoprotein (LDL)-Cholesterol (LDL-C), triglyceride (TG), and total cholesterol (TC) levels.

Separate serum samples were stored at −80 °C until thawed for analysis. Serum levels of phosphatidylethanolamine (PtdEtn) 16:0/22:6, PlsEtn 16:0/22:6, PlsEtn 18:0/20:5, and PlsEtn 16:0/22:4 were determined using stable isotope dilution liquid chromatography tandem mass spectrometry (LC-MS/MS) using ^13^C-PlsEtn 16:0/22:6 and ^13^C-PtdEtn 16:0/22:6. 10 µL of internal standards (1 µg/mL), 40 µL of 4% formic acid in water and 0.5 mL of 100% ethyl acetate (EtOAc) were added to 20 µL of serum. The mixture was vortexed for 15 min and centrifuged at 4 °C for 2 min at 1400 rpm. The supernatant was used for the analysis of each sample. All extracts were stored at −80 °C until analysis. 100 µL of the EtOAc fraction was injected by flow injection at 600 µL/min and ionized using negative atmospheric pressure chemical ionization (APCI) mode using a triple quadrupole mass spectrometer (IONICS 3Q, Ionics Mass Spectrometry Group, Bolton, ON, Canada) coupled to a Gilson GX-271 (Gilson Inc., Middleton, WI, USA). Parent (M–H): fragment (sn2 fatty acid) transitions were monitored for each analyte in the multiple reaction monitoring (MRM) mode. Standard curves were generated using ^12^C-PlsEtn 16:0/22:6 and ^12^C-PtdEtn 16:0/22:6 and encompassed the full biological range observed, *r*^2^ > 0.98. All standards and stable isotopes were >95% pure and manufactured by Phenomenome Discoveries Inc., (Saskatoon, SK, Canada).

A Plasmalogen Biosynthesis value (PBV) was created for each participant by calculating and combining three PlsEtn/PlsEtn and two PlsEtn/PtdEtn ratios. As explained below, the PlsEtn16:0/22:6 to PtdEtn16:0/22:6 and PlsEtn18:0/20:5 to PtdEtn16:0/22:6 ratios were used to assess the relative PlsEtn and PtdEtn pool sizes; and the PlsEtn16:0/22:6 to PlsEtn16:0/22:4, PlsEtn18:0/20:5 to PlsEtn16:0/22:4, and PlsEtn 18:0/20:5 to PlsEtn16:0/22:6 ratios were used to assess peroxisomal β-oxidation. These molecules were selected based on their sn-2 fatty acids. These fatty acids are either metabolized by peroxisomal β-oxidation (22:4 and 22:6) [32] or are a product of peroxisomal β-oxidation (20:5 or 22:6) [33,34] and are also known to be associated with AD [35,36]. PlsEtn and PtdEtn species used in these ratios are among the most abundant PlsEtn and PtdEtn species in the blood [37], thus, PlsEtn to PtdEtn ratio represents their relative abundance [38]. For the calculation of ratios, each ratio was divided by its sex-specific mean, and then log10 transformed to equally weight each ratio, after which these five values were averaged to create the PBV.

### 2.4. APOE Genotyping

Participants were genotyped for *APOE* alleles by Polymorphic DNA Technologies. Participants were grouped according to *APOE* genotype as follows: ε2ε3 (*n* = 149), ε3ε3 (*n* = 745), and ε3ε4 or ε4ε4 (ε3ε4/ε4ε4, total *n* = 269). Participants with an *APOE* genotype of ε2ε2 (*n* = 4) or ε2ε4 (*n* = 16) were excluded from the analyses.

### 2.5. Statistical Analyses

In descriptive analyses, males and females were compared using chi-square or Fishers’s exact tests for categorical variables and Wilcoxon rank-sum tests for continuous variables. To reduce skewness, TG, TC, HDL-C, LDL-C, HDL ratio (HDL-R) (HDL-C/TC) were log10 transformed. For continuous outcomes, such as GCOG, we first screened potential predictors by examining correlations with the outcome and then fit multiple linear regression models including both variables of interest (PBV, HDL-R, *APOE* ε2ε3, *APOE* ε3ε4/ε4ε4) with terms to adjust for age, sex, and education. The predictors that passed the screening were then included in a single multiple regression model, with terms for age, sex, and education. For continuous predictors, associations are shown per standard deviation (SD) of the predictor. We augmented the model by including the interactions of *APOE* genotypes with PBV; we also repeated linear regression models in stratified *APOE* ε2ε3, *APOE* ε3ε3, and *APOE* ε3ε4/ε4ε4 groups. For categorical variables, we used a similar approach: we first ran a Pearson chi-square or a Fisher’s Exact test to determine the presence of association with various covariates, followed by multiple logistic regression. For the determination of associations with AD in the presence of MCI, ordinal logistic regression was performed. Results are reported as Odds Ratio (OR) or Coefficient (coef), *p* value. Stata 13.1 was used to perform all analyses; statistical significance is *p* < 0.05 in all analyses.

## 3. Results

### 3.1. Demographics

Participant demographic characteristics and the number of people in each diagnostic category are given in Table 1 and Table 2.

### 3.2. Relation of PBV and APOE to AD and Level of Cognition

The relation of PBV and *APOE* to the diagnosis of Alzheimer’s disease (AD) and mild cognitive impairment (MCI) versus no cognitive impairment (NCI) was examined using an ordered logistic regression model. After adjusting for serum lipids and demographic measures, a higher serum PBV level was associated with a lower odds of MCI/AD (Table 3). Specifically, each 1 SD increase in PBV was associated with about a 30% reduced odds of MCI/AD. By contrast, an *APOE* ε2ε3 was associated with about a 50% lower odds of MCI/AD, and an *APOE* ε3ε4/ε4ε4 was associated with nearly twice the odds of MCI/AD (Table 3).

Because clinical diagnoses represent cutoffs across a continuum of cognition, we next examined the relation of PBV and *APOE* to GCOG using a linear regression model. After adjusting for serum lipids and demographic measures, each 1 SD higher serum PBV level was associated with more than a 10% higher GCOG (Table 3). *APOE* ε2ε3 also was associated with more than a 10% higher GCOG, whereas *APOE* ε3ε4/ε4ε4 was associated with more than a 20% lower GCOG (Table 3).

Because GCOG represents a collection of somewhat dissociable cognitive domains, we next conducted a series of linear regression models to examine the relation of PBV and *APOE* to the level of function in five cognitive domains; episodic memory, visuospatial ability, semantic memory, perceptual speed, working memory. Interestingly, PBV was associated with better function in all five cognitive domains with its strongest effects on episodic memory (Table 3). Similarly, *APOE* ε3ε4/ε4ε4 was associated with lower function in all five domains with its strongest effects on episodic memory (Table 3). By contrast, *APOE* ε2ε3 was associated with better performance on tests of episodic memory and semantic memory (Table 3).

**Figure 1:**
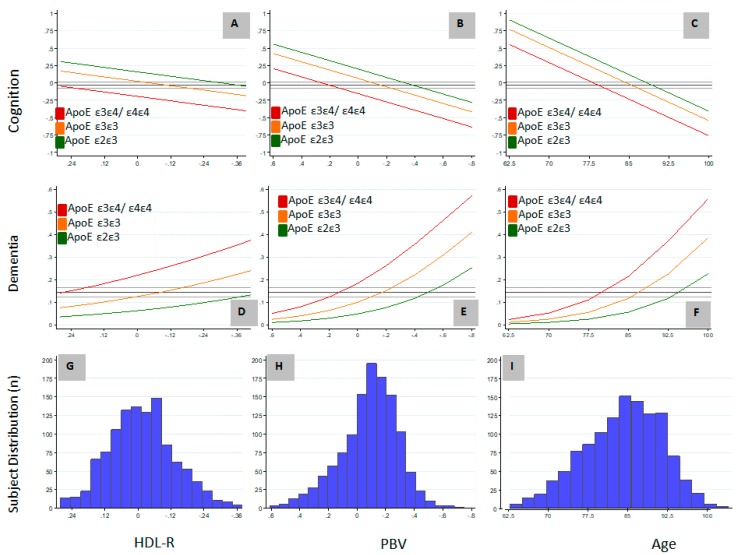
The association of the distribution of High Density Lipoprotein (HDL) ratio (HDL-R), Plasmalogen Biosynthesis value (PBV) and age with probability of dementia and cognition in Apolipoprotein E (APOE) ε2ε3, ε3ε3 and ε3ε4/ε4ε4 carriers. (**A**) Effect of HDL-R on cognition in different APOE genotypes; (**B**) Effect of PBV on cognition in different APOE genotypes; (**C**) Effect of age on cognition in different APOE genotypes; (**D**) Effect of HDL-R on probability of dementia in different APOE genotypes; (**E**) Effect of PBV on probability of dementia in different APOE genotypes; (**F**) Effect of age on probability of dementia in different APOE genotypes; (**G**) Distribution of HDL-R in the study cohort; (**H**) Distribution of PBV in the study cohort; (**I**) Distribution of age in the study cohort. Multiple regression analysis was carried out to determine the association of the distribution of HDL ratio, PBV, and age with dementia and cognition in the study cohort. Cognition was measured as global cognition score (GCOG), which is the average of *z*-scores from a battery of cognitive measurements. Mean normalized, log10 transformed values of HDL-R, PBV, and mean-centered values of age are in the *x*-axes. *y*-axes represent GCOG in the graphs depicting the associations of cognition with HDL-R (A), PBV (B) and age (C); in the graphs depicting dementia (D–F), *y*-axes represent the probability of dementia as a fraction. In the distribution graphs (G–I), *y*-axes represent the number of individuals. HDL-R: HDL-C to total cholesterol ratio; PBV: Plasmalogen Biosynthesis value.

Of the variables determined to be independently associated with AD and cognition in this study, only HDL-R and PBV are potentially modifiable. We included HDL-R in our analyses based on the association of HDL-C [39] and TC [40] with AD, and also because of the association of *APOE* lipoprotein with HDL particles [41]. The relationship between these variables and the non-modifiable variables of age and *APOE* genotype on GCOG and the probability of dementia are graphically represented in Figure 1. The study population was normally distributed with respect to age, HDL-R, and PBV (Figure 1G–I) and the *APOE* ε2ε3//ε3ε3//ε3ε4/ε4ε4 genotype distribution (13/64/23, Table 2) was similar to other published distributions [3]. The mean study GCOG was -0.03 and the overall study probability of dementia was 14.5%. If cognition and dementia were considered as a continuum resulting from aging, relative to the *APOE* ε3ε3 genotype, the association of an *APOE* ε3ε4/ε4ε4 genotype with cognition or dementia was approximately equal to being older by 6.2 and 6.9 years, respectively, and a *APOE* ε2ε3 genotype was approximately equal to being younger by about 3.9 and 7.5 years of age, respectively (Figure 1C,F). Although a higher HDL-R is associated with higher cognition and a lower probability of dementia (Figure 1A,D), large differences in HDL-R are required to offset the associations attributable to *APOE* genotype. In contrast, relatively small differences in PBV are needed to offset the associations attributable to *APOE* genotype (Figure 1B,E). For example, the probability of dementia in *APOE* ε3ε4/ε4ε4 carriers with a PBV 0.3 units higher was equal to that of *APOE* ε3ε3 carriers, and with a PBV 0.6 units higher it was equal to that of *APOE* ε2ε3 carriers. Considering that 0.6 units represents less than 50% of the observed 1.3 unit PBV range, the biological system underlying PBV, if pharmacologically modifiable, has the potential to have a disease-modifying effect equal to or greater than the *APOE* genotype.

**Figure 2:**
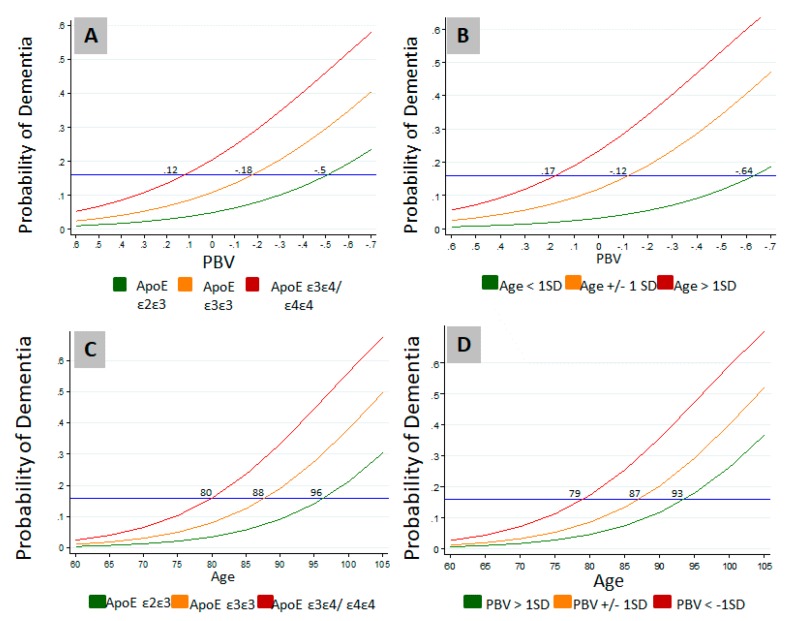
Effects of age and *APOE* genotype on the probability of dementia is modulated by PBV. (**A**) Modulation of the effect of genotype on the probability of dementia by PBV; (**B**) Effects of age distribution on the probability of dementia at different PBV levels; (**C**) Effects of genotype on the probability of dementia at different ages; (**D**) Effects of PBV distribution on the probability of dementia at different ages. Logistic regression was carried out to determine the effects of PBV, age, and APOE genotype on the probability of dementia in the study cohort. PBV: Plasmalogen Biosynthesis Value; SD: Standard Deviation.

Modulation of the effects of age and *APOE* genotype on the probability of dementia by PBV is further illustrated in Figure 2. Probability of dementia approached zero irrespective of the genotype when the PBV index is numerically higher (Figure 2A). Similarly, while higher age increased dementia probability, the probability of dementia approached zero irrespective of the age at higher PBVs (Figure 2B). These results suggest that higher PBV is protective even when other risk factors are present. As expected, another well-known protective factor, the presence of *APOE ε2ε3* allele, appeared to be also shielding against the effect of age on the probability of dementia (Figure 2C). Consequently, *APOE ε2ε3* lowered the probability of dementia by an age equivalent to approximately 16 years compared to *APOE* ε3ε4/ε4ε4 carriers (Figure 2C). In comparison, higher PBV (>1 SD), was protective against the effect of age on the probability of dementia by an age equivalent to approximately 14 years compared to lower PBV (< 1 SD) (Figure 2D).

### 3.3. Study Variable Associations on Serum Lipids

The associations between the study variables and serum lipids are described in Table 4. Higher PBV was associated with lower TG, higher TC, higher LDL-C, higher HDL-C, and a higher HDL-R. A *APOE* ε2ε3 genotype was associated with lower LDL-C and a higher HDL-R, and a *APOE* ε3ε4/ε4ε4 genotype was associated with higher TC, higher LDL-C, and a lower HDL-R. No association between *APOE* genotype and PBV was observed. Of the study variables investigated, only PBV and *APOE* were associated with HDL-R levels (Table 4).

## 4. Discussion

Age and *APOE* genotype are the primary variables associated with the probability of AD and the observations of this study support these well accepted facts. The importance of understanding the biochemical pathway(s) upon or within which advancing age or the ApoE isoforms interact to effect changes in cognition and cause AD cannot be overstated. As an inborn genetic risk factor, *APOE* genotype is, de facto, within the causative cascade of AD. The ApoE isoform association with disease prevalence (ε4 > ε3 > ε2) [3] is analogous to a medicinal chemistry structure–activity relationship and suggestive of a quantitative and continuous underlying mechanism. The isoform-specific prevalence rates further suggest that if this mechanism were to be found and successfully targeted, that AD prevalence could be reduced to at least that of the ε2ε3 genotype. This would result in an overall reduction in AD cases by 75% or more [42].

The two prevailing ApoE hypotheses involve interactions with amyloid and/or cholesterol regulation and homeostasis and a third, less studied mechanism involves altered membrane composition. All three are interrelated biochemically. ApoE has been shown to exert an isoform-specific causal effect on amyloid accumulation in both imaging [43,44] and post-mortem [45,46] studies, and membrane PlsEtn enrichment has been shown to dose-dependently decrease Aβ_1–42_ via increased α-secretase [47]. However, the hypothesis that amyloid accumulation causes a decline in cognition remains unproven. To the contrary, anti-amyloid therapies have consistently failed to affect cognition in humans [48].

Age is the primary variable associated with the probability of AD and the observations of this study support this well-accepted fact. The distributions of age, cognitive status, and diagnosis of AD within this study allowed us to robustly investigate the associations between non-modifiable variables, such as age and *APOE* genotype, and putatively modifiable metabolic covariates with AD and cognition. After correcting for *APOE* genotype and demographic variables, biomarkers of PBV and the ratio of HDL-R were independently associated with cognition and dementia with PBV having a magnitude of association similar to that of *APOE* genotype.

Although no direct interactions between PBV and *APOE* were observed, both the protective *APOE* ε2ε3 genotype and high (protective) levels of PBV were associated with higher HDL-R (Table 4) and higher HDL-R was independently associated with higher cognition and a lower odds of dementia (Table 3). Since HDL-R is a measure of HDL-MCE, these observations suggest that both *APOE* and PlsEtn may be interacting with HDL-MCE via independent mechanisms.

The role of cholesterol dysregulation and *APOE* in AD has been recently reviewed [18,19]. Higher levels of free cholesterol in human post-mortem cortical membranes has been associated with lower cognition [49]; lower total cholesterol in the Cerebrospinal fluid (CSF) of AD patients is suggestive of lower cholesterol efflux in AD [50,51]; and increasing membrane cholesterol reportedly induces the β-secretase pathway and the accumulation of amyloid independent of *APOE* genotype [52]. *APOE* has been shown to exert an isoform-specific causal effect on amyloid accumulation in both imaging [43,44] and post-mortem [45,46] studies, and membrane PlsEtn enrichment has been shown to dose-dependently decrease Aβ_1–42_ via increased α-secretase [47]. However, the hypothesis that amyloid accumulation *causes* a decline in cognition remains to be proven clinically [48].

The clinical implications of this study are obvious. This is the first reported evidence of a metabolic phenotype with the same clinical characteristics as the *APOE* ε2ε3 genotype. The probability of dementia in participants with either a high PBV or an *APOE* ε2ε3 genotype was indistinguishable. Accordingly, effective pharmacological targeting of the plasmalogen biosynthesis pathway has the potential of having a disease-modifying effect in AD equal to or greater than that of the *APOE* ε2ε3 genotype.

## 5. Conclusions

The key conclusions of this study are that lower serum PBV levels are associated with higher odds of MCI/AD and with lower levels of cognition. Higher PBV levels (>1 SD) appeared to modulate the negative effects of age and APOE ε4 allele on the probability of dementia. This study, thus, identifies lower levels of PBV as a potentially modifiable risk factor for dementia. This finding would, potentially, pave the way for interventions that can lower the risk of dementia in high-risk individuals.

## Figures and Tables

**Table 1:** Demographic and serum lipid summary by gender.

	Female (*n* = 921)		Male (*n* = 284)		
**Age, Education**					
**Variable**	**Mean**	**SD**	**Mean**	**SD**	***p*-Value**
**Age (range)**	83.8 (58.3–103.9)	7.6	84.3 (64.2–101.0)	7.1	3.8 × 10^−1^
**Education**	15.5	3.4	16.1	4.0	1.7 × 10^−2^ *
**Serum Lipids**					
**Variable**	**Mean**	**SD**	**Mean**	**SD**	***p*-Value**
**TG**	136.8	67.9	122.7	59.2	3.3e × 10^−3^ *
**TC**	192.6	38.3	161.7	35.6	6.0 × 10^−28^ *
**HDL-C**	63.9	18.4	52.5	15.2	4.9 × 10^−18^ *
**LDL-C**	101.4	33.2	84.6	29.1	1.9 × 10^−12^ *
**HDL-R**	0.338	0.092	0.331	0.088	3.5 × 10^−1^
**Serum Ethanolamine Glycerophospholipids**					
**Variable**	**Mean**	**SD**	**Mean**	**SD**	***p*-Value**
**PE226**	1.60	0.89	1.08	0.63	4.6 × 10^−19^ *
**PL224**	0.85	0.48	0.72	0.31	8.0 × 10^−5^ *
**PL205**	1.35	1.83	0.98	0.81	1.1 × 10^−3^ *
**PL226**	3.48	2.28	2.84	1.22	8.5 × 10^−6^ *
**Plasmalogen Ratios**					
**Variable**	**Mean**	**SD**	**Mean**	**SD**	***p*-Value**
**PL205/PE226**	0.65	0.55	0.715	0.480	7.3 × 10^−2^
**PL226/PE226**	1.81	0.88	2.18	1.00	1.6 × 10^−9^ *
**PL205/PL224**	1.94	2.49	1.67	1.98	1.0 × 10^−1^
**PL226/PL224**	4.55	2.36	4.39	2.27	2.9 × 10^−1^
**PL205/PL226**	0.36	0.23	0.337	0.186	1.4 × 10^−1^
**PBV**	−0.09	0.21	−0.075	0.179	2.9 × 10^−1^

Mean and standard deviation of the demographic variables, lipoproteins and phospholipid are given per gender. Serum lipid values are given in mg/dL. Internal standard–normalized values are given for ethanolamine glycerophospholipids and plasmalogen ratios. TG: Triglycerides, TC: Total cholesterol; HDL-C; HDL cholesterol; LDL-C: LDL cholesterol, HDL-R: HDL cholesterol to Total cholesterol ratio; PE226: Phosphatidyl ethanolamine 16:0/22:6; PL224: Plasmalogen ethanolamine 16:0/22:4; PL205: Plasmalogen ethanolamine 18:0/20:5; PL226: Plasmalogen ethanolamine 16:0/22:6; PL205/PE226: ratio of Plasmalogen ethanolamine 18:0/20:5 to Phosphatidyl ethanolamine 16:0/22:6; PL226/PE226: ratio of Plasmalogen ethanolamine 16:0/22:6 to Phosphatidyl ethanolamine 16:0/22:6; PL205/PL224: ratio of Plasmalogen ethanolamine 18:0/20:5 to Plasmalogen ethanolamine 16:0/22:4; PL226/PL224: ratio of Plasmalogen ethanolamine 16:0/22:6 to Plasmalogen ethanolamine 16:0/22:4; PL205/PL226: ratio of Plasmalogen ethanolamine 18:0/20:5 to Plasmalogen ethanolamine 16:0/22:6; PBV: Averaged value of the five mean normalized (gender-based) log10 transformed plasmalogen ratios. For clarity, significant *p* values (*p* < 0.05) are indicated by an asterisk *.

**Table 2:** Clinical diagnoses and global cognition in key variable classes.

Clinical Diagnosis and Cognition by Key Variable Classes							
Variable Class	All	No Dementia	Dementia (%)	NCI	MCI	AD	GCOG Mean (SD)
**Female**	921	784	137(14.9)	597	168	104	−0.025 (0.829)
**Male**	284	246	38 (13.4)	184	68	26	−0.051 (0.756)
**PBV > 1 SD**	183	172	11 (6.0)	143	26	9	0.238 (0.647)
**PBV ± 1 SD**	851	740	111 (13.0)	556	177	80	−0.243 (0.782)
**PBV < 1 SD**	171	118	53 (31.0)	82	33	41	−0.374 (0.996)
**ApoE e2e3**	149	139	10 (6.7)	116	24	3	0.178 (0.589)
**+ PBV > 1 SD**	25	25	0 (0)	23	2	0	0.370 (0.442)
**+ PBV ± 1 SD**	106	100	6 (5.7)	82	18	2	0.184 (0.612)
**+ PBV < 1 SD**	18	14	4 (22.2)	11	4	1	−0.139 (0.528)
**ApoE e3e3**	745	641	104 (14.0)	480	152	75	−0.028 (0.817)
**+ PBV > 1 SD**	122	115	7 (5.7)	96	17	5	0.262 (0.606)
**+ PBV ± 1 SD**	511	448	63 (12.3)	330	113	42	−0.019 (0.771)
**+ PBV < 1 SD**	112	78	34 (30.4)	54	22	28	−0.406 (1.063)
**ApoE e3e4/e4e4**	269	210	59 (21.9)	151	55	51	−0.181 (0.900)
**+ PBV > 1 SD**	28	24	4 (14.3)	18	6	4	−0.010 (0.917)
**+ PBV ± 1 SD**	206	164	42 (20.4)	119	43	36	−0.173 (0.881)
**+ PBV < 1 SD**	35	22	13 (37.1)	14	6	11	−0.390 (1.007)

Clinical diagnosis (NCI, MCI, and AD) and global cognition score (GCOG) according to gender, PBV levels and APOE allele composition. Note lesser number of AD cases and higher GCOG when PBV > 1 SD. SD: Standard deviation; NCI: No cognitive impairment; MCI: Mild cognitive impairment; AD: Alzheimer’s disease; PBV: Plasmalogen Biosynthesis Value.

**Table 3:** Multivariate association of clinical and biological variables with dementia, mild cognitive impairment/Alzheimer’s disease (MCI/AD), and cognition.

	Dementia	NCI-MCI-AD	Global Cognition	Episodic Memory	Visuospatial Ability	Perceptual Speed	Semantic Memory	Working Memory
	**(*n* = 1085)**	**(*n* = 1030)**	**(*n* = 1049)**	**(*n* = 1026)**	**(*n* = 992)**	**(*n* = 1005)**	**(*n* = 1022)**	**(*n* = 1049)**
**Variable**	**OR ^3^ (*p*)**	**OR ^2^ (*p*)**	**Coef. ^1^ (*p*)**	**Coef. ^1^ (*p*)**	**Coef. ^1^ (*p*)**	**Coef. ^1^ (*p*)**	**Coef. ^1^ (*p*)**	**Coef. ^1^ (*p*)**
**PBV**	0.607 (3.3 × 10^−6^) *	0.699 (2.5 × 10^−6^) *	0.118 (1.7 × 10^−7^) *	0.134 (5.3 × 10^−6^) *	0.051 (4.4 × 10^−2^) *	0.111 (5.2 × 10^−5^) *	0.107 (3.1 × 10^−5^) *	0.079 (1.6 × 10^−3^) *
**HDL-R^4^**	0.773 (5.9 × 10^−3^) *	0.904 (1.6 × 10^−1^)	0.062 (5.6 × 10^−3^) *	0.078 (8.5 × 10^−3^) *	0.018 (4.8 × 10^−1^)	0.019 (4.8 × 10^−1^)	0.042 (1.0 × 10^−1^)	0.060 (1.6 × 10^−2^) *
***APOE* ε3ε3**	Reference	Reference	Reference	Reference	Reference	Reference	Reference	Reference
***APOE* ε2ε3**	0.442 (3.0 × 10^−2^) *	0.462 (1.8 × 10^−3^) *	0.136 (4.2 × 10^−2^) *	0.230 (8.1 × 10^−3^) *	0.062 (4.1 × 10^−1^)	−0.033 (5.2 × 10^−5^) *	0.112 (1.4 × 10^−1^)	0.048 (5.2 × 10^−1^)
***APOE* ε3ε4, ε4ε4**	2.125 (2.2 × 10^−4^) *	1.996 (1.9 × 10^−5^) *	−0.217 (6.2 × 10^−5^) *	−0.319 (7.6 × 10^−6^) *	−0.076 (2.2 × 10^−1^)	−0.276 (3.1 × 10^−5^) *	−0.190 (2.0 × 10^−3^) *	−0.105 (7.9 × 10^−2^)
Age *	2.271 (1.4 × 10^−13^) *	2.056 (2.0 × 10^−19^) *	−0.262 (4.1 × 10^−3^) *	−0.290 (2.6 × 10^−22^) *	−0.137 (8.6 × 10^−8^) *	−0.352 (1.6 × 10^−35^) *	−0.191 (1.0 × 10^−13^) *	−0.177 (1.9 × 10^−12^) *
Education *	0.957 (6.5 × 10^−1^)	0.917 (2.3 × 10^−1^)	0.183 (4.8 × 10^−16^) *	0.165 (1.9 × 10^−8^) *	0.255 (1.0 × 10^−22^) *	0.204 (1.2 × 10^−13^) *	0.229 (6.7 × 10^−19^) *	0.174 (3.2 × 10^−12^) *
**Female**	Reference	Reference	Reference	Reference	Reference	Reference	Reference	Reference
**Male**	1.035 (8.8 × 10^−1^)	1.104 (5.5 × 10^−1^)	−0.048 (3.7 × 10^−1^)	−0.159 (2.3 × 10^−2^) *	0.205 (6.9 × 10^−4^) *	−0.156 (1.6 × 10^−2^) *	−0.013 (8.2 × 10^−1^)	0.066 (2.7 × 10^−1^)

Multiple regression analysis was carried out to determine the associations of plasmalogen biosynthesis value (PBV), HDL ratio (HDL-R), APOE genotype, age, education and gender with odds of dementia (logistic regression), MCI/AD diagnosis (logistic regression) and cognitive scores (linear regression). ^1^ Coefficients for continuous variables expressed per standard deviation (SD); multiple regression; ^2^ Odds ratios for continuous variables expressed per SD, ordinal logistic regression with AD/MCI/NCI as the outcome; ^3^ Odds ratios for continuous variables expressed per SD, multiple logistic regression; ^4^ HDL-R was mean normalized and log_10_ transformed; ^5^ Age and Education were mean centered. For clarity, significant *p* values (*p* < 0.05) are indicated by an asterisk *.

**Table 4:** Multivariate association of clinical and biological variables with serum lipids.

	Total Cholesterol (*n* = 1085)	LDL-Cholesterol (*n* = 1078)	HDL-Cholesterol (*n* = 909)	HDL-R (*n* = 909)	Trigylcerides (*n* = 909)
**Variable**	**Coef. ^1^ (*p*)**	**Coef. ^1^ (*p*)**	**Coef. ^1^ (*p*)**	**Coef. ^1^ (*p*)**	**Coef. ^1^ (*p*)**
**PBV**	0.009 (5.7 × 10^−4^) *	0.014 (2.6 × 10^−3^) *	0.029 (3.8 × 10^−15^) *	0.020 (5.0 × 10^−8^) *	−0.043 (3.8 × 10^−14^) *
***APOE* ε3ε3**	Reference	Reference	Reference	Reference	Reference
***APOE* ε2ε3**	−0.009 (2.8 × 10^−1^)	−0.036 (7.6 × 10^−3^) *	0.018 (9.7 × 10^−2^)	0.027 (1.3 × 10^−2^) *	0.014 (4.2 × 10^−1^)
***APOE* ε3ε4, ε4ε4**	0.021 (9.7 × 10^−4^) *	0.042 (1.2 × 10^−4^) *	−0.002 (8.1 × 10^−1^)	−0.023 (6.5 × 10^−3^) *	0.002 (8.8 × 10^−1^)
**Age**	−0.009 (1.1 × 10^−3^) *	−0.014 (2.0 × 10^−3^) *	−0.002 (5.6 × 10^−1^)	0.007 (6.1 × 10^−2^)	−0.006 (2.6 × 10^−1^)
**Education**	−0.0004 (8.8 × 10^−1^)	0.0001 (9.9 × 10^−1^)	0.003 (3.8 × 10^−1^)	0.004 (3.2 × 10^−1^)	−0.008 (1.4 × 10^−1^)
**Female**	Reference	Reference	Reference	Reference	Reference
**Male**	−0.001 (8.5 × 10^−1^)	−0.001 (9.4 × 10^−1^)	−0.002 (8.1 × 10^−1^)	−0.001 (8.7 × 10^−1^)	0.007 (6.3 × 10^−1^)

Multiple regression analysis was carried out to determine the associations of plasmalogen biosynthesis value (PBV), *APOE* genotype, age, education, and gender with serum lipid parameters. ^1^ Coefficients for continuous variables expressed per standard deviation (SD); Age and Education were mean centered; Lipids were mean normalized then Log_10_ transformed. For clarity, significant *p* values (*p* < 0.05) are indicated by an asterisk *.
